# Limitations in the Effect of Screening on Breast Cancer Mortality

**DOI:** 10.1200/JCO.2018.78.0270

**Published:** 2018-09-04

**Authors:** Anna-Belle Beau, Per Kragh Andersen, Ilse Vejborg, Elsebeth Lynge

**Affiliations:** Anna-Belle Beau, Per Kragh Andersen, and Elsebeth Lynge, University of Copenhagen; Ilse Vejborg, Copenhagen University Hospital, Rigshospitalet, Copenhagen, Denmark

## Abstract

**Purpose:**

Randomized, controlled trials showed that screening reduces breast cancer mortality rates, but some recent observational studies have concluded that programmatic screening has had minor effect on breast cancer mortality rates. This apparent contradiction might be explained by the use of aggregated data in observational studies. We assessed the long-term effect of screening using individual-level data.

**Materials and Methods:**

Using data from mammography screening in the Copenhagen and Danish national registers, we compared the observed breast cancer mortality rate in women invited to screening with the expected rate in absence of screening. The effect was examined using the “naïve model,” which included all breast cancer deaths; the “follow-up model,” which counted only breast cancer deaths in women diagnosed after their first invitation to screening; and the “evaluation model,” which is similar to the follow-up model during screening age, but after screening age, which counted only breast cancer deaths and person-years in women diagnosed during screening age.

**Results:**

We included 18,781,292 person-years, 976,743 of which were from women invited to screening. The naïve and follow-up models showed, respectively, 10% and 11% reduction in breast cancer mortality after invitation to screening. However, many breast cancer deaths occurred in women whose cancer was diagnosed when they were no longer eligible for screening. Accounting for this dilution, the evaluation model showed a 20% (95% CI, 10% to 29%) reduction in breast cancer mortality after invitation to screening.

**Conclusion:**

Screening had a clear long-term beneficial effect with a 20% reduction in breast cancer–associated mortality in the invited population. However, this effect was, by nature, restricted to breast cancer deaths in women who could potentially benefit from screening. Our study highlights the complexity in evaluating the long-term effect of breast cancer screening from observational data.

## INTRODUCTION

Breast cancer screening’s primary aim is to reduce the rate of breast cancer mortality. In the 1980s, several randomized controlled trials, first from Sweden, showed that screening with mammography only could help reduce breast cancer mortality rates.^1^ In one recent review, the combined evidence from the randomized controlled trials showed that screening delivered about a 20% reduction in breast cancer mortality rate^2^; another review found that screening reduced the mortality rate associated with breast cancer by an average of 25%^3^; and another found there was a mortality reduction across all ages varying from 12% in women aged 39 to 49 years, 14% in women aged 50 to 59 years, 33% in women aged 60 to 69 years, and 20% in women aged 70 to 74 years.^4^

On this background, screening has been widely implemented in routine health care. Nevertheless, breast cancer screening is one of the most intensively debated health care interventions. During the same period that screening was introduced, breast cancer treatment improved, which makes it difficult to separate the effect of screening from the effect of treatment. To address this problem, modeling studies based on US data concluded that both screening and treatment helped reduce the rate of death from breast cancer.^5^

Recent studies have focused on the incidence of late-stage breast cancer. The underlying assumption was that the effect of screening should result in a decrease in the incidence of late-stage breast cancer. If not observed, a decline in the breast cancer mortality rate would be attributable to treatment and not to screening. A study from the United States concluded that “the reduction in breast cancer mortality after the implementation of screening was predominantly the result of improved systemic therapy,”^6^ and another study, using Dutch data, concluded that the “screening program would have had little influence on the decrease in breast cancer mortality.”^7^ These studies analyzed data for all women above screening age, which is 40 years in the United States and 50 years in the Netherlands. However, not all women in these road age groups could have been affected by screening, and such studies, therefore, cannot correctly capture the possible effect of screening. For a proper evaluation of screening, it is necessary to avoid the use of broad age groups, to focus instead on the actual birth cohorts of women potentially affected by screening, and to use individual records instead of aggregated data.

In this study, we used individual-level data to analyze the long-term effect on breast cancer mortality rates of a population-based breast cancer screening program in Copenhagen, Denmark.

## MATERIALS AND METHODS

### Breast Cancer Screening Program

The Copenhagen screening program started with biennial screening on April 1, 1991. Women aged 50 to 69 years were invited every second year to screening. Other regional, organized programs were implemented in Funen in 1993, in Frederiksberg in 1994, in Bornholm in 2001, and in part of Vestsjælland in 2004. Nationwide, organized screening in Denmark started at the end of 2007 and early 2008.

### Study Participants

#### Contemporary groups (screening period).

**Fig 1.**
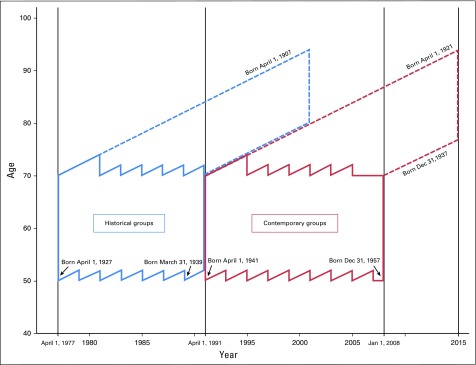
Study design illustrated in Lexis diagram.

The Copenhagen study group included women invited to screening in the Copenhagen program between April 1, 1991 and December 31, 2007. The regional control group included women living in a nonscreening region (the rest of Denmark excluding Copenhagen, Funen, Frederiksberg, Bornholm, and Vestsjælland) during the same period. In the regional control group, a first pseudo-invitation date was allocated to each woman following the scheme similar to that of the Copenhagen study group. Contemporary groups are illustrated in the Lexis diagram (Fig 1).

#### Historical control groups (before screening).

The historical Copenhagen control group included women living in Copenhagen between April 1, 1977 and March 31, 1991. The historical, regional control group included women living in a nonscreening region during the same period. For both groups, first pseudo-invitation dates were allocated similarly to the procedure used for the regional control group. The construction of the groups is detailed in Data Supplement.

Using data from mammography screening in Copenhagen and Danish national registers,^8-10^ we compared the observed breast cancer mortality rate in women invited to screening with the expected rate in absence of screening.^11,12^ Data sources and the statistical analysis are described in the Data Supplement.

### The Naïve Model

The naïve model included all breast cancer deaths occurring during the follow-up period (thus disregarding date of diagnosis and assuming that all breast cancer cases contributed to breast cancer deaths, even the cases diagnosed before first [or pseudo] date of invitation to screening). Person-years were accumulated from date of invitation (or pseudo-invitation) until date of death, emigration, or end of follow-up, whichever occurred first. However, many breast cancer cases would be diagnosed before the woman had been invited to screening. Thus, in the naïve model, the reduction in breast cancer mortality rate is diluted by breast cancer deaths occurring in patients who could not have benefited from screening.

### The Follow-Up Model

The follow-up model, as described by Nyström et al,^13^ included only breast cancer deaths derived from breast cancer cases diagnosed after the first/pseudo-invitation to screening. Person-years were accumulated from date of invitation (or pseudo-invitation) until date of death, emigration, or end of follow-up, whichever occurred first. However, in this model, a nonnegligible proportion of breast cancer deaths would derive from cases diagnosed after the women had left screening. Thus, the effect of screening is diluted by breast cancer deaths occurring in patients diagnosed after screening age.^14,15^

### The Evaluation Model

Another approach to avoid the dilution phenomenon is to evaluate the effect of screening only among breast cancer cases diagnosed during the screening period. We estimated a modified version of the evaluation model developed by Nyström et al.^13^ We included breast cancer deaths occurring among women who received a breast cancer diagnosis during the screening period (ie, during the screening age plus 6 months, to allow time for diagnosis) and equivalent for the control groups. Accordingly, we ensured that only breast cancer deaths that could potentially be affected by screening were counted. For women of screening age, person-years were accumulated from date of invitation (or pseudo-invitation) until date of death, emigration, or end of follow-up, whichever occurred first. For women after screening age, person-years were accumulated only among women with breast cancer diagnosed during the screening period. In doing so, we ensured that only the women at risk for developing the event according to our definition contributed person-years in the analysis. Until end of screening age, the follow-up and the evaluation models were identical, but the two models differed after end of screening age. There are no empirical data to unambiguously fill the gap between these two models.

Analyses were conducted using SAS, version 9.4 (SAS Institute, Cary, NC). The study was approved by the Danish Data Protection Agency (No. 2015-57-0121).

## RESULTS

The study was based on 18,781,292 person-years of data, among which 976,743 were from women invited to screening and 17,804,549 were from control subjects. The individual average follow-up time from invitation (or pseudo-invitation) to end of follow-up was 11.6 years. The mean age at first/pseudo-invitation was 56.9 years.

### The Naïve Model

The crude breast cancer mortality rate in the Copenhagen study group was 130.0 per 100,000 person-years, compared with 148.2 per 100,000 person-years estimated in the absence of screening. The age-adjusted rate ratio was 0.90 (95% CI, 0.84 to 0.97; Data Supplement).

### The Follow-Up Model

Figure 1 illustrates the dilution phenomenon. Across all ages, only 67% of breast cancer deaths expected after first invitation to screening in the study group occurred among women diagnosed with breast cancer after screening started; in the age group 50 to 54 years, the percentage was only 23%.

The crude breast cancer mortality rate in the Copenhagen study group was 85.4 per 100,000 person-years, compared with 100.7 per 100,000 person-years estimated in the absence of screening. The age-adjusted rate ratio was 0.89 (95% CI, 0.82 to 0.98; Table 1).

**Table 1.**
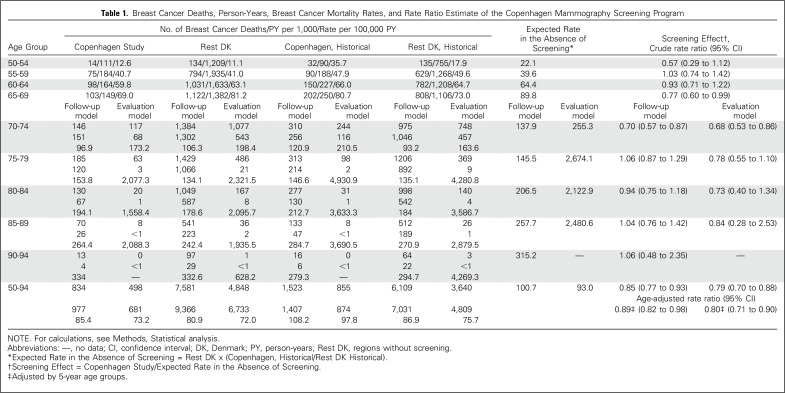
Breast Cancer Deaths, Person-Years, Breast Cancer Mortality Rates, and Rate Ratio Estimate of the Copenhagen Mammography Screening Program

### The Evaluation Model

**Fig 2.**
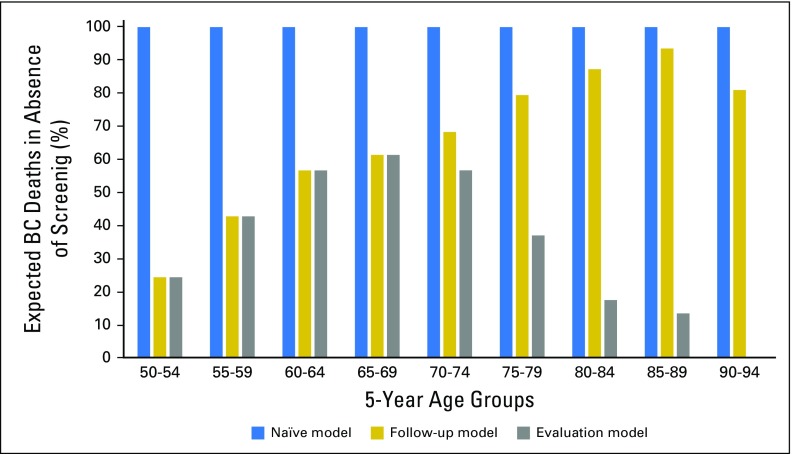
Percentage of expected breast cancer deaths in the contemporary Copenhagen study group using the naïve model as the reference model. Naïve model: Cases were all breast cancer deaths occurring during the follow-up period; person-years were accumulated from all the women during the follow-up period. Follow-up model: Cases were breast cancer deaths occurring among women who received a breast cancer diagnosis after the first pseudo-invitation to screening; person-years were accumulated from all the women during the follow-up period. Evaluation model: Cases were breast cancer deaths occurring among women who received a breast cancer diagnosis during the pseudoscreening period; person-years were accumulated after the pseudoscreening period only among women with breast cancer that was diagnosed during the pseudoscreening period.

As the length of follow-up after screening age increased, the amount of dilution by breast cancer deaths among cases diagnosed after the last screen increased. Overall, only 43% of breast cancer deaths expected after first invitation to screening in the study group occurred among women diagnosed when eligible for screening (Fig 2).

The number of breast cancer deaths and accumulated person-years decreased from age ≥ 70 years. The crude breast cancer mortality rate in the Copenhagen study group was 73.2 per 100,000 person-years as compared with 93.0 per 100,000 person-years expected in the absence of screening. This resulted in a reduction in breast cancer mortality after invitation to screening. The age-adjusted rate ratio was 0.80 (95% CI, 0.71 to 0.90; Table 1).

**Fig 3.**
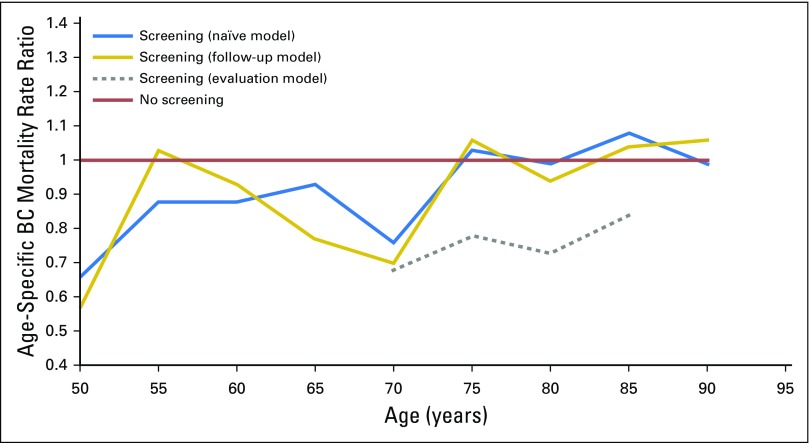
Age-specific breast cancer (BC) mortality rate ratios. The area surrounded by the gray line represents the screening age (50 to 69 years). Naïve model: Cases were all BC deaths occurring during the follow-up period; person-years were accumulated from all the women during the follow-up period. Follow-up model: Cases were BC deaths occurring among women who received a BC diagnosis after first invitation to screening; person-years were accumulated from all the women during the follow-up period. Evaluation model: Cases were BC deaths occurring among women who received a BC diagnosis during the screening period; person-years were accumulated after the screening period only among women with BC that was diagnosed during the screening period.

In the naïve and follow-up models, the effect of screening was restricted to the screening age; there also was a marked effect of screening after the end of the screening age in the evaluation model (Fig 3).

## DISCUSSION

We conducted a population-based study using individual records investigating the long-term impact of breast cancer screening on breast cancer mortality rates. According to our study data, a substantial proportion of breast cancer deaths were among women whose breast cancer was diagnosed when the women were no longer eligible for screening. Inclusion of these breast cancer deaths in the analysis will inevitably dilute the measured effect of screening. When the analysis was restricted to the women who could have benefited from screening, our data showed a 20% reduction in breast cancer mortality after invitation to screening.

### Comparison With Other Studies

Studies based on broad age groups showed no marked effect of screening on breast cancer mortality.^6,7^ In these studies, it was not possible to separate breast cancer deaths among women invited to screening from those among noninvited women. Hence, a substantial proportion of breast cancer deaths included in these analyses will be among women who received a breast cancer diagnosis when they were not eligible for screening (ie, either before the start or after the end of screening age), as seen also from Dalarna, Sweden.^16^ In contrast, in cohort studies with individual records, it is possible to separate women invited to screening from those not invited, and to link data from each woman to cancer and cause of death registries. Then, the analyses could be restricted to women and breast cancer cases that could potentially have benefited from screening.

The concept of the follow-up model and the evaluation model were first presented by Nyström et al^13^ in their overview of the Swedish randomized controlled trials. They found invitation to screening was associated with a 15% reduction in breast cancer mortality rate when the follow-up model was used, and a 21% reduction when using the evaluation model. These results are in line with ours, indicating the importance of dilution in long-term follow-up data. Therefore, in later updates, Nyström et al.^17^ (relative risk, 0.85; 95% CI, 0.73 to 0.98) and Tabár et al^18^ (relative risk, 0.69; 95% CI, 0.56 to 0.84) reported only results from the evaluation model. In the latter study, less than half of prevented breast cancer deaths were observed within the first 10 years of follow-up.

In the Swedish analyses,^13^ the evaluation model used the person-years from all women from randomization until the end of the follow-up. In our evaluation model, we restricted the person-years after screening age to the women diagnosed with breast cancer during screening age. This choice was motivated by the fact that in the evaluation model, only these women were at risk for dying of breast cancer after end of screening. It should be noted that this calculation will give high mortality rates in all groups after the end of screening.

Previously, the Copenhagen screening program was analyzed using the follow-up model for the first 10 years after the program started. The analysis showed a 25% (95% CI, 11% 37%) reduction in breast cancer mortality rates after invitation to screening.^19^ After a maximum of 23 years of follow-up since the start of the program, the reduction in breast cancer mortality rate was, as estimated from the follow-up model, only 11% (95% CI, 2% to 18%). This difference was a result of the increasing dilution with time from breast cancer deaths in women who received a diagnosis after screening age. Hence, a long follow-up after end of screening permits evaluation of the long-term impact; however, the longer the postscreening follow-up, the greater the dilution.^20^ US mortality data from 2007 to 2011 showed that approximately one-third of breast cancer deaths in women were attributed to diagnoses after the age of 70 years.^21^ García-Albéniz et al^22^ highlighted the problems in using observational data in evaluation of cancer screening outcomes. Their data set was different from the one used in the current study. Where García-Albéniz et al^22^ compared colorectal cancer incidence between persons undergoing and not undergoing colonoscopy, we compared breast cancer mortality between populations offered and not offered screening mammography and adjusted for prescreening differences between these two populations. Thus, we avoided bias from selective participation and confounding from differences between geographical areas and/or time periods. García-Albéniz et al^22^ demonstrated the importance of never classifying screening status retrospectively; accordingly, screening status was classified only prospectively in our study.

Furthermore, the potential impact of breast cancer screening on breast cancer mortality rates depends also on the coverage by examination (the number of participating women divided by the number of targeted women). A substantial proportion of the targeted population must be screened for screening to be effective in reducing mortality rates associated with breast cancer at the population level. In the Copenhagen program, the coverage by examination at first invitation was between 73% for the first targeted birth cohorts and 64% for the last birth cohorts.^23^ A drop in coverage was observed with the increasing invitation number.^23^ These differences in coverage are expected to have an impact on the outcome of breast cancer screening. Then, the true effectiveness of breast cancer screening might be underestimated.

### Limitations and Strengths

Screening advances the date of diagnosis, and slow-growing and less aggressive tumors, or even nonaggressive tumors in terms of overdiagnosis, are more likely to be detected by screening, so the survival advantage of screening detection can be artificially inflated in the study group as compared with the control subjects. However, we expected the lead time bias to be limited in our evaluation model, because the number of deaths resulting from breast cancer and person-years would be affected by it. Screening, in particular the first screen, may lead to diagnosis of slow-growing tumors. But this potential time bias was not expected to affect our data set seriously, because most women attended screening several times, and all patients with breast cancer were followed up for breast cancer deaths for a minimum of 10 years. Moreover, the evaluation model relied on the fact that cases of breast cancer deaths were previously registered with a diagnosis of breast cancer. This was verified for 96% of the breast cancer deaths.

The breast cancer mortality rates in our follow-up model increased with age, similar to the pattern seen in routine breast cancer mortality statistics. The data in the follow-up model, however, do not give a proper presentation of a possible screening effect because the majority of the breast cancer deaths after the age of 70 years were diagnosed after the women stopped being offered screening. This problem was overcome in the evaluation model by including after the age of 70 years only breast cancer deaths in women whose cancer was diagnosed when they were still offered screening. It should be noted, however, that the breast cancer mortality rates in our evaluation model increased very rapidly after the age of 70 years. This may intuitively look strange, but it is explained by the fact that women diagnosed with breast cancer prior to the age of 70 years are at a very high risk of dying of breast cancer after the age of 70 years. This is true for women offered screening and women not offered screening. The rates in the evaluation model, therefore, should be used only for internal comparisons between the study group and three control groups.

The strengths of our study include the use of individual data with linkage between the exposure and outcome for each woman. Another strength of our study is that the study group included all women offered screening and not only those who attended screening. In this way, we avoided self-selection bias. In addition, this study took advantage of the use of three control groups; hence, the expected breast cancer mortality rate in the absence of screening was estimated by the rate in a nonscreening region, controlling for regional differences in a prescreening period. Both region and period were thus controlled for, but it was assumed that in the absence of screening, the breast cancer mortality rate changed identically over time in screening and nonscreening regions. It means no interaction between region and period (ie, no unsynchronized changes between screening and nonscreening regions). This assumption was considered realistic because, in Denmark, diagnostics and treatment of breast cancer have been organized nationwide since 1977.^24^ This assumption was further supported by regional trends in breast cancer mortality rates in the prescreening period.^25^ Last, our study was based on a long follow-up, with a 17-year screening period and 20 years of postscreening time. All patients with breast cancer were followed up for breast cancer–related mortality for a minimum of 10 years, ensuring a long follow-up time even for cases diagnosed at an early stage. Women screening positive in our study group underwent the same diagnostic procedures as women with symptoms of breast cancer.

Our findings highlight the complexity of evaluating the long-term impact of screening on breast cancer mortality rates. We found that screening had a clear, long-term, beneficial impact of a 20% reduction in breast cancer mortality rate in the invited population. Nevertheless, this effect of screening is restricted to breast cancer deaths in women who could potentially benefit from screening. As women age, a rapidly increasing proportion of breast cancer deaths occur in women who received a breast cancer diagnosis after screening age. If this dilution is not adequately addressed, the effect of screening is inevitably underestimated. Thus, screening is clearly beneficial but, after screening age, only for a diminishing proportion of women.
